# Food Waste on Foodservice: An Overview through the Perspective of Sustainable Dimensions

**DOI:** 10.3390/foods10061175

**Published:** 2021-05-24

**Authors:** Maísa Lins, Renata Puppin Zandonadi, António Raposo, Veronica Cortez Ginani

**Affiliations:** 1 Post-Graduate Program in Human Nutrition, University of Brasília, Brasília 70910-900, Brazil; 2 Department of Nutrition, University of Brasília, Brasília 70910-900, Brazil; renatapz@unb.br (R.P.Z.); vcginani@gmail.com (V.C.G.); 3 CBIOS (Research Center for Biosciences and Health Technologies), Universidade Lusófona de Humanidades e Tecnologias, Campo Grande 376, 1749-024 Lisboa, Portugal

**Keywords:** food waste, meal production, sustainability metrics

## Abstract

Food waste (FW) is a current, complex, and widely debated issue in various spheres of society. Globally, about 2.6 trillion dollars per year is lost because of wasted food. Part of FW is preventable, and it is necessary to identify where it occurs. In most cases, FW occurs at the end of the production chain (meal preparation and distribution). Identifying the main food service failures on FW is important for developing efficient strategies for reducing them. Therefore, this study aimed to perform a narrative review of the impacts caused by FW in food services considering the three dimensions of sustainability (social, economic, or environmental). Multiple reasons were identified in this review that impacts those three dimensions, such as the cost of wasted raw material, use of cleaning material, the energy consumption, salary of food handlers, the water footprint, the amounts of rest-intake, production waste, energy density wasted, use of organic food, and food donation. Identifying these aspects can contribute to reduce FW impacts for better sustainable development, develop tools to measure FW, and assist food service managers in minimizing FW.

## 1. Introduction

Food waste (FW) is a multifaceted and complex issue. It integrates sustainable production, which is also a continuous and multidimensional process and covers several parameters [1]. Studies and quantification of waste are hampered by various concepts and definitions on the subject, with no universal consensus [2,3].

Although there are numerous definitions in the scientific literature for FW, standardization is necessary to understand and study this subject. A review showed some of the most used terms and their definitions [4]. The term “food loss” generally refers to post-harvest losses; loss of food (wheter it is safe or not safe for human consumption) that is discarded by some “defect”; or the unintentional food disposal that occurs before the meal’s production [5,6]. The term “surplus food” is related to the excesses produced, but not ingested by any human, even under correct conservation conditions [7,8]. The definition of “food waste” includes “food loss” and “surplus food” [6,9].

Sachs [10] highlighted that FW influences three sustainability pillars (economic, social, and environmental) and one must seek the population’s quality of life in regard to environmental balance to transform the current production form into sustainable development [10]. However, the potential impacts caused by FW from food services considering the sustainability dimensions were not well explored by literature. The Food and Agriculture Organization (FAO) [5] estimated the worldwide cost of FW to be close to 2.6 trillion dollars of losses per year, considering the sustainability dimensions (about 700 billion dollars of environmental costs, a trillion in the economic scope, and 900 billion in the social scope).

The impact of FW from the final phase of the food production chain stands out because of several costs along with the meal production, including non-renewable energy sources, food handlers’ salaries, use of products to clean the area and sanitize vegetables and fruits, among others [11]. A study demonstrated that 60% of food is discarded in consumption and food services are responsible for 34% of this discard [12].

Considering that FW squanders many natural resources (impacting sustainability) mainly at the end of the food production chain, food recovery could prevent surplus food from being disposed of in a landfill [13], providing new food uses, such as feeding insecure populations. A narrative review was performed to identify the technical, regulatory, and social context relationships between food recovery and food safety in U.S. foodservice settings [13]. The authors identified the additional steps in the foodservice process that stem from food recovery (increased potential for cross-contamination and hazard amplification due to temperature variations; the potential risk factors, transmission routes, and major hazards). The hazard identification step could provide strategies to best manage food safety hazards in recovery in foodservice settings. However, insufficient data and unclear regulatory guidelines are barriers to implementing food-recovery-safe food service practices [13].

It is a great challenge to develop and find studies that measure the social, environmental, and economic impacts of waste within foodservice. Some factors must be considered throughout the food production impacting the economy, environment, and health of those who eat meals outside or at home. In a recent systematic review by Dhir et al. [14], the authors identified 58 studies related directly to FW in the profit sector of hospitality and food services (HaFS). They highlighted a critical research gap related to analysis among these studies, such as the narrow focus of prior studies, underestimating the quantity of waste, limited geographic scope, limited breadth of analysis in terms of mediating and moderating variables, and lack of theoretical frameworks. The authors indicated some potential research areas, such as advances in quantification methods to measure FW, utilization of diverse research methods and variables, deeper diagnoses of interventions and nudges to create awareness about FW, and improved theoretical perspectives. Exploring the three sustainability dimensions involving FW in the food sector can contribute to deepen the subject and fill this gap. The present study observes this gap and poses unprecedented aspects that must be discussed and investigated in greater detail.

Therefore, it is necessary to identify where and how waste occurs to minimize it, being this the primary goal of the present review. We hypothesize that the knowledge about social, economic, and environmental impacts of FW can help develop strategies to reduce it, strengthen local sustainability, and highlight the importance of public policies that encourage sustainable actions in restaurants. A recent review [15] reaffirms the need for a more comprehensive approach to foodservice FW, suggesting that it is necessary to face the problem from a holistic view. Hence, considering the FW on foodservice through the perspective of sustainable dimensions, it is possible to see the problem in its entirety. In this sense, this study aimed to review the literature on the impacts caused by FW in food services to show the enormity of variables that are impacted by FW in the three dimensions of sustainability, emphasizing the importance of developing tools that make it possible to measure them and assist food service managers to minimize FW and its impacts.

## 2. Methods

This narrative review was performed following three steps: conducting the search, reviewing abstracts and full-texts, and discussing results. For this, the PubMed, EM-BASE, Scopus, Science Direct, Web of Science, Science Direct, and Google Scholar databases were searched to identify the relevant studies, according to the development of the review. The final search was conducted in October 2020 and included English language-based international articles, online reports, and electronic books. The keyword “food waste” was used combined with other terms such as restaurant, foodservice, school canteens, school food programs, leftover, economic impact, social impact, environmental impact, or sustainability. After the complete search, the abstracts were read to ensure that they address the topic of interest. All duplicates were removed, and the abstracts of the remaining articles were reviewed to ensure that they address the review inclusion criteria. The eligible criteria were studies that analyzed FW in at least one of the three sustainability dimensions (social, economic, or environmental). Therefore, the studies of interest focusing on foodservice, FW, and sustainable dimensions were summarized and synthesized to integrate the narrative review. Since it is a narrative review, it was unnecessary to document the literature search on specific platforms [16].

## 3. Theoretical Background

Sustainable production uses resources conscientiously to maximize the useful life of products, minimize the environmental, social, and even economic costs of production, and reuse products whenever possible in other production chains [16]. Thus, for sustainable production to exist, actions in three dimensions must be focused on the economic, environmental, and social. According to Sachs [17], it is necessary to seek the population’s quality of life in regard to environmental balance to integrate the current production type with sustainable development. In addition, there is a lack of public policies that guide and support actions to combat FW in several countries [18]. It should be noted that the 2030 Agenda defined by the United Nations—which consists of an action plan for governments, individuals, and companies from all countries—has among its objectives the reduction of waste and the reversal of the negative scenario generated by this incoherent production today. There are 17 sustainable development goals (SDG) and 169 targets for all countries to adopt according to their priorities. Some of the objectives of this plan are to encourage the conscious consumption and production of food, with targets that motivate the efficient use of natural resources, management, purchasing, and sustainable practices, in addition to the sustainable management of chemical products [19].

Three targets of Goal 12 of the 2030 Agenda, directly related to the reduction of waste generation, are highlighted in this research: (i) the reduction of FW at the retail and consumer level and the reduction of food loss in the planting and post-harvest process; (ii) decrease in waste production through prevention, reduction, recycling, and reuse; (iii) promoting sustainable public procurement practices, following national policies and priorities [19].

An example of an initiative to reduce the amount wasted is the Zero Hunger/Zero Waste project, which aims to distribute produced and uneaten food to vulnerable people in the United States. The project is widely encouraged by a private company and involves several partner companies across the country. However, this project’s primary goal is to bring the amount wasted in the institutions down to zero, meaning an effective and efficient production [20].

The reduction of FW is a concrete global goal but initially lacks evaluations and strategies to measure costs, impacts, and the main points of waste generation for it to be effective [21]. Thus, it is essential to understand the impact of waste at each stage of the production chain and what factors contribute most to FW.

It is known that losses occur in the entire food production chain, but waste is most perceived in the final phase, where the most significant impacts are generated since, in the processing and distribution, there are several investments added to the food. In these final stages, labor for production and energy, among other procedures that frequently use non-renewable energy sources, such as liquefied petroleum gas used by production stoves, must be considered [11,22].

A survey conducted in Brazil shows that approximately 60% of food is discarded at the time of consumption, with collective food services responsible for 34% of this discard [11,12]. In this sense, several surveys are carried out in food services to quantify waste and the results confirm this percentage but do not identify precisely the impact caused [3,12,23,24,25,26,27,28]. 

Although the volume of FW is easily measured during all stages of the production chain, it results from a complex set of factors with consequences in different spheres. In addition to the amount of food discarded, the direct and indirect impacts on society, the economy, and the environment are of great concern. Estimates indicate a loss of approximately 2.6 trillion dollars a year, considering economic, social, and environmental aspects worldwide. Some examples of waste impacts are the consumption of fuel and energy for storage and transportation, the low quality of life of individuals in the case of hunger, and the direct values of food purchase [5].

## 4. The Food Waste Generation and Impacts

### 4.1. Food Services

A study analyzed in-depth some possible strategies for reducing FW and loss in food services. The authors revealed that meat losses and waste worldwide are around 5% [28]. In monetary terms, this waste is over 20%, equivalent to US$122.00 per capita/year. Regarding fruits and vegetables, although slightly lower but no less worrisome, the figure reaches US$108 per capita/year. For cereals, the value reaches US$78.00 per capita/year [28]. 

However, monetary value is relatively easy to obtain. When we realize that the amount and costs of wasted food in the world today would be sufficient to feed up to 2 billion people, we understand that there is a greater impact behind it. More than double the number of hungry individuals worldwide could be fed. If there were a correct distribution of what is produced, no individual worldwide would go hungry [29].

Studies have been carried out in food services to quantify waste [30,31,32,33]. A systematic review was carried out in the United States to describe methods used to measure FW and respective results in the National School Lunch Program (NSLP) across time, considering post-consumer waste. The authors identified four methods, including in-person visual estimation (n = 11), digital photography (*n* = 11), direct weighing (*n* = 23), and a combination of in-person visual estimation, digital photography, and/or direct weighing (*n* = 8). Fruits and vegetables were the products that stood out in the studies due to the enormous waste volume. The studies were characterized by their design being transversal or post-intervention [32]. Another study considered the pre-consumer waste in Colorado schools and showed that the waste in this stage is very low. Most of the waste reason was spoilage, expiration, and/or contamination [33]. 

In another survey performed in different food services from Finland, such as daycare centers, schools, and company canteens, the establishments recorded the amount of food discarded for two weeks. The information was transferred to a research database, with 51 restaurants participating in the study. The results revealed an average of 17.5% of prepared foods directly thrown in the trash. Of these, 2.2% were discarded during preparation (kitchen waste), distribution leftover/serving loss were 11.3%, and 3.9% of customers discarded after serving (rest-intake/plate leftover) [30]. A recent review shows the need for a more comprehensive approach to the impact of FW [15]. The research refers to the use of the circular economy as a way to facilitate the discussion on FW by all the actors involved. The authors mention the need to face the restaurants’ FW problem from a holistic view [15], which opens the opportunity to discuss it considering the three sustainability dimensions in our review.

### 4.2. Economic Dimension

Understanding the economic dimension is crucial to encourage reductions in this generation of waste in food production [34,35]. The company must be built and managed in such a way that the equipment and tools used to bring financial returns with a balance concerning the other two pillars. For this, it is necessary to identify all aspects related to the control of the impact generated by FW in this dimension.

The cost of wasted raw material is essential. It reveals the financial value spent on food that has been discarded. It is estimated that almost US$750 billion in food is thrown away each year worldwide, considering only the cost of ingredients [36]. Because it is so easily perceived, this direct cost is widely observed by managers and restaurant owners and can be a way to reduce FW and, consequently, financial losses [36]. 

However, other items must be considered concerning the economic dimension, such as expenses with acquiring products, energy, transportation, distribution, processing, and labor. Thus, the cost involved is much higher than that perceived superficially with the acquisition of the product [23,37].

Bianco et al. [38] highlighted that a critical sustainable indicator is electricity expense. This study performed in Italy in the hotel sector estimates that it may be possible to achieve 13% energy savings in the country between 2017 and 2030. The hotel only implemented energy efficiency measures, such as replacing current lamps with LED lamps [38].

Adopting electric energy policies is essential for reducing direct values in the food services’ electricity bill. There are energy rationing options that can often be detrimental to the quality of the products offered. For example, turning off the freezer at night to save energy puts all stored food at risk and is an inappropriate strategy for food quality [39]. That is why energy efficiency strategies are more appropriate, unlike rationing. These measures seek to produce with lower energy demands while maintaining the quality of what is produced. It is possible to improve efficiency by replacing equipment, optimizing operations, and investing in training [39].

Evaluating the economic dimension, there is also the labor required for meal production, which is an indispensable aspect to be considered. Hours worked must also be considered as an economic impact of meal production. Di Maria et al. [40] highlighted that this is an indispensable aspect in assessing the impact generated by FW. The value of labor is directly related to waste (since the salary paid to the food handler for the time employed to perform the task) is discarded along with the despised production [40]. 

The cost of paying food handlers is directly related to the FW since their salary is discarded with the time spent to produce the meals/foods thrown in the trash. Identifying the aspects involved in FW can allow specific corrections in the most significant impact items. Thus, investments in training and equipment in food production are essential since the food scraps and the quality of the meal produced are also associated with the handlers’ patterns.

**Figure 1:**
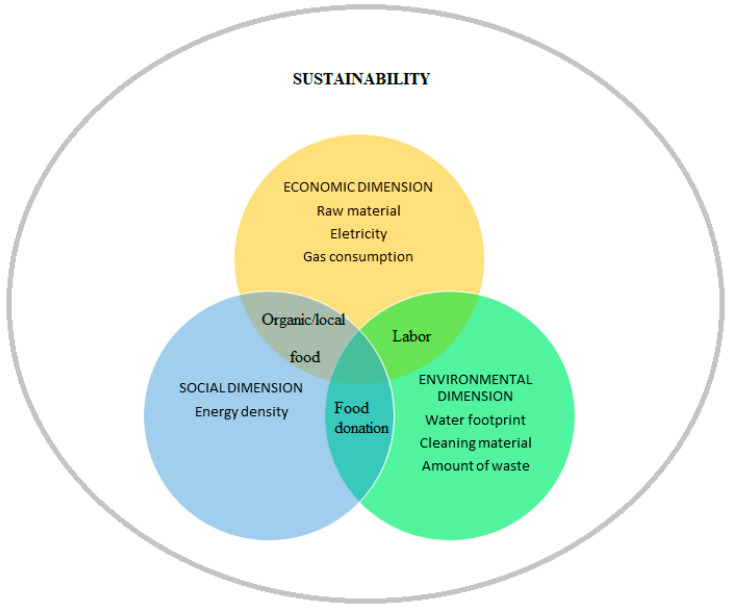
Impacts of food waste in foodservice.

It is important to highlight that one item can impact more than one sustainability dimension (Figure 1). However, we opt to divide in this review to facilitate comprehension considering the dimension in which it was most influential according to the studies.

### 4.3. Environmental Dimension

In addition to the economic impact of FW, the environmental issue is usually the most discussed on sustainability. It is related to the reduction of polluting gases, minimization of waste, and reuse of surplus production. One of the main ways of verifying the impacts generated by the waste in this dimension is the amount wasted, in volume or weight. This waste can impact the soil and the greenhouse effect with polluting gases due to organic waste accumulation. The accumulated environmental damage can be irreversible and damage several future generations [8,41,42]. 

As shown by Papargyropolou et al., restaurant employees often do not handle the food correctly due to lack of attention or do not receive the necessary training, increasing the waste rate [43]. The lack of adequate equipment or structure for meal production can also be a reason for this waste during manipulation; another reason is the workers’ overload; overwork can lead the handler to exhaustion and decrease his food preparation commitment [43,44]. 

Besides the food wasted during the production/distribution, which is the rest-intake, distribution leftover, and production leftover, there is more to consider [44]. The excess of discarded food, often directed to landfills, produces polluting gases and heavy metals that can remain in the environment for up to 100 years after the extinction of these garbage “deposits” [45]. These expended resources impact the environment, with data showing about 3.3 billion tons of carbon emitted [45,46]. This type of garbage disposal from production units is presented as the most unwanted possibility in the hierarchy from the United States Environmental Protection Agency (U.S. EPA) [47,48].

The U.S. EPA determines that it should be preferred to reduce the amount of waste at the top of the hierarchy. The second option in the scale of preference is to donate food to hungry people. If those first are not possible, the food should be used to feed animals rather than providing energy to industrial uses. The two least preferred options are composting and discarding the food in landfills or incineration [48].

It is also worth mentioning the necessary care with food preservation to minimize losses due to deterioration. Products in unwanted patterns characterize this situation (such as crushed or moldy fruit, expired products, and undistributed surplus production). Losses can come from inadequate planning, supplier selection, improper reception, or failures during pre-preparation, preparation, and distribution [44,49,50].

It is observed that, environmentally, the damage caused by the accumulation of garbage in landfills can result in the scarcity of natural resources, emphasizing water and reducing biodiversity. Consequently, in some locations, it makes it impossible for most species to exist. Therefore, waste is a problem that must be treated seriously and considered in all its scope and urgency [5].

FW analysis must also consider the waste of water used in the entire food production process (from planting to consumption) and the water footprint (WF) is one of the main possibilities for assessing water consumption [51]. Some studies that evaluate the WF of meal production indicate that animal origins’ food use needs to be reviewed. Diets based on products of plant origin allow a considerable reduction in the impact of the social dimension [52,53,54,55,56].

These studies show that it is essential to assess the menu’s primary food source (animal or vegetable). However, it is also important to understand that analyzing the WF is not enough to make changes to the menu. It is necessary to understand the acceptance of plant-origin foods in relation to those of animal origin. If the waste of plant foods is considerably greater than animal products, the impact may be greater. Thus, the impacts and changes necessary to reduce the impact generated by FW should be evaluated, considering all variables [57,58].

Another aspect is cleaning materials. When misused, they become inefficient and make the investment in this type of product in vain. They also cause water pollution due to incorrect dilution and foodborne diseases, as evidenced by Roche [59]. Untreated chemicals are often discarded and contaminate rivers and streams, which in some cases remain in the environment and contaminate the food chain, being harmful to human health [59,60]. Detergents, for example, if discarded in excess in the environment, produce a layer in the water that prevents the entry of oxygen for the breathing of living beings in the aquatic ecosystem. Although laws require biodegradable products, it is fairly common for this rule not to be practiced in some countries. Moreover, the variety of cleaning products contains chlorine, heavy metals, and various chemical compounds. These products, when combined, enhance their damage and can affect the ecosystem. Even people who consume fish have been exposed to contaminated water, for example [61]. Thus, it reinforces the idea that actions should not be isolated, e.g., considering only a single aspect of waste [62]. To minimize food rejection, Buzby and Guthrie argued that interventions aimed at the acceptability and presentation of dishes are crucial [63].

### 4.4. Social Dimension

The social dimension can be perceived internally and externally. In this dimension, management responsibilities and good practices with the company’s employees are considered and the community’s impacts with attitudes that reduce hunger and encourage local commerce, for example. A healthy, organized work environment must be guaranteed, with adequate remuneration and actions that value and encourage the populations’ well-being and environment [64,65]. 

Thus, knowledge about good production practices is essential. Good production practices are understood to be the set of practices that must be adopted from the selection and purchase of raw materials and products used to produce meals minimizing the incidence of foodborne diseases [66]. Examples of these practices prioritize using products that expire in a shorter period than those with greater durability. Another aspect is the appreciation of local product suppliers, also avoiding more significant emissions of gases in long-distance transport and encouraging regional trade [44,49,50].

The factors that influence the generation of waste are numerous. However, information on the exact causes for this excess is limited [2,25,67]. Studies presented the food recovery hierarchy with different desirable levels of generation/destination of FW [49,68]. The donation of food to people in situations of vulnerability or groups of need is the U.S.EPA hierarchy’s second level [68,69]. 

The incorrect management of food production and distribution is inconsistent with peoples’ situation of vulnerability and hunger in the world. Data from the World Food Program (2019) indicate that more than 820 million people are still hungry globally, while a third of everything produced each year is thrown away [29]. If proper access to food was guaranteed, countless people could be fed and well-nourished. In other words, waste is also an important public health problem. Its reduction is essential to enable any strategy to be developed to feed the growing world population, sustainably and equitably [29,70,71].

Another essential step in the production chain that can directly affect society is food production and distribution management. Despite seeming (and being) enough to feed everyone, the current systems’ food is inaccessible to around 11% of the world population [70]. 

It is noteworthy that the food wasted worldwide would be enough to feed up to 2 billion people, more than double the number of hungry people in actual society. While a third of what is produced is unusable, thousands of people do not have access to safe and quality food. If there was a correct distribution of what is produced, no individual would go hungry [8,29,41,42,72].

Thus, it is necessary to highlight that all individuals’ physical and economic access to food in quantity and quality sufficient to supply their physiological needs for a healthy life and food preferences is a human right [73]. Food and nutritional security (FNS) consist in realizing the right of everyone to regular and permanent access to quality food, in sufficient quantity, without compromising access to other essential needs, based on health-promoting food practices that respect cultural diversity and are socially, economically, and environmentally sustainable [74].

From that moment on, it became the public’s duty to adopt policies that promote and guarantee food security for the population [74]. Availability and access in sufficient quantity and quality to maintain human health and good nutrition must be guaranteed by FNS and are essential for individuals and nations’ well-being [75].

It should also be noted that estimates indicate that by 2100, more than 10.8 billion people will live on planet Earth [76]. This world population will demand an increase of approximately 70% in food production, making it essential to reverse the existing waste scenario [77].

The social dimension also stands out for the destination of the surplus of food suitable for human consumption. Since FW is considered to be any part of food that is discarded, regardless of its potential content of compounds retaining a high value, depending on its origin, it can contain a variable chemical composition of carbohydrates, proteins, lipids, and other components that could be used in human and animal feeding [78]. Food donation is authorized in some countries and should be considered, as it is an important social factor, considering the data already presented on the correlation between FW and world hunger. Targeting safe food to people with restricted access to quality food is extremely important. In this way, the food surplus will not be destined for landfills or other conventional disposal types that can also affect the environment. In addition, the social importance of this conduct is highlighted, which keeps food in the production chain and improves the lives of people in need [72,79,80].

The donation of prepared foods is allowed in some countries, but food service managers are concerned with the distribution of surplus food to vulnerable people [81]. Restaurants that send leftover meals to people in need are responsible for food safety and must ensure these meals’ hygienic quality [81].

In some countries, food donation initiatives are being used to allocate leftover food so that it is not discarded as waste and is used, once it is suitable for consumption. Some countries allow the donation of food for people, families, or groups of vulnerable individuals. Food must be within the expiration date and safe hygienic and sanitary conditions for human consumption must be maintained [44,82,83,84,85]. This strategy proved to be important for various levels of society impacted by actions to target leftover food, especially people in need, food handlers, donors, and the community society [86]. However, many food services choose not to donate food because they are concerned with punishments generated by non-conformities in this destination [87]. 

Another motivation that prevents food donation, in some cases, is that the food may not incorporate the habits of the population for which it is intended and, for this reason, be neglected and waste occurs [88]. It may be that the scarcity of research in developing countries, for example, that identifies the possibilities of making production more sustainable is a reason for the absence of public policies [85]. 

A solution regularly adopted for discarded food that is in unsafe conditions for human consumption is composting. This technique is used in vegetable gardens to produce biofuel, animal feed, and even power generation in small home installations. In other words, FW, if handled correctly, can be converted into benefits by increasing recycling rates [89,90,91].

It is possible to identify some actions involving the directing of foodservice waste for composting. An action carried out for six months in a hospital restaurant transformed food disposal into quality fertilizer and identified a reduction of approximately six tons of solid waste that would be sent to the landfill [92]. 

However, a study pointed out that there is still much resistance in the food sector to adhere to initiatives that could reduce FW’s environmental impact [93]. The justification for this reluctance is the cost, clients’ resistance, and the lack of employees’ training to properly implement more sustainable tools or techniques. [93].

The reduction of waste makes it possible to optimize the use of land and agricultural resources that could be used for other functions, such as the production of food for the hungry [94]. The use of agricultural land and other limited resources such as water will also need to be increased to produce enough food for everyone. It is necessary to devise ways to offer nutritionally adequate food with less environmental, social, and economic impacts, and, above all, it is necessary to reduce waste [95]. 

It is reinforced that, usually, the problems that impact society are due to avoidable waste. They result from planning failures at all stages of the production chain. It is a problem that, if circumvented, will contribute to mitigating different social issues such as a greater offer of jobs, since there will be more land suitable for planting and a lower rate of people who demand health services [46,96,97].

### 4.5. Waste Prevention–Possibilities to Decrease the Impact

The search for measures capable of making production systems more sustainable, including reducing the costs generated by FW, must be a commitment to the whole society. It is necessary to develop effective public policies for waste management, dependent on quantifying FW generated in all sectors [91]. 

An important alternative to prevent FW concerning the mentioned variables is menu planning [85]. From well-executed planning, considering the target audience, quality of the raw material, operational capacity of the team, and structure, among others, it is possible to increase the acceptance of meals and reduce waste [85,92]. Another aspect is to use strategies such as improving the dishes’ presentation, carrying out nutritional education work, and adjusting the portion sizes [85]. Consequently, production will be more efficient and profitable, with the acquisition of better-quality raw materials and rational use of energy [82,83,85]. 

Food acceptance/rejection is associated with taste, presentation/appearance, portioning, texture, temperature, among other aspects [98]. A study highlights that poor planning and overproduction are among the main reasons for the high amount of food discarded in food services [30]. Buzby and Guthrie [63] argue that interventions aimed at acceptability and presentation of dishes are crucial to minimizing food rejection. It is important to emphasize that reaching the ideal acceptability goal must respect some criteria so that the meal does not constitute a potential risk for its consumers [62,99]. 

Another publication points out that it is necessary to know the public and identify consumers’ food preferences so that the preparations are well accepted [100]. It is essential to teach children from the early school years about “food waste” [63,99]. Corroborating these studies, the ReFed Restaurant Guide shows some critical points to minimize FW in food services. Among them is menu design, portion size choices, wasting tracking and analytics, inventory management, and production planning, among others [101]. Food and nutrition education activities (which improve the perception of diners about a balanced diet and, consequently, the served meal acceptance) must be constant. One option to reduce waste is to present incentives for those who do not leave leftovers [85].

All studies presented are relevant to elucidate the need to develop new studies involving all dimensions of sustainability. Many aspects have been presented and analyzed many times individually. It is important to highlight that the three sustainability dimensions can interfere with each other and, therefore, further studies involving all of them (together) must be conducted in food services. This could be a step towards greater awareness of the problem of FW in different types of food services. Thus, the adequate dimensioning of the problem can be achieved, initially, through educational actions that elucidate the collective benefits generated with FW control.

In the food service sector, there is no consensus on the necessary measures to avoid food waste [102]. The lack of conceptual alignment of practices to avoid FW may reflect the lack of understanding of what FW represents. Thus, the need to disclose social, economic and environmental dimensions equally is reinforced once again so that effective measures are thought and executed [102].

### 4.6. Limitations

It is important to highlight that the nature of the narrative reviews presents some limitations as the assumptions and the planning are not often known; the scope is limited by the defined query, search terms, the selection criteria, and evaluation biases not known; and narrative reviews are not reproducible [103]. However, narrative reviews have an important role in continuing education because they provide readers with up-to-date knowledge about a specific topic or theme [104,105].

## 5. Conclusions

In this narrative review, we described the impacts caused by FW in food services based on the three pillars of sustainability. In the tertiary sector, specifically in food services, combating waste actions are essential given the large number of meals prepared. Although there are several strategies to minimize the cost of FW, there are numerous causes that influence this impact. The causes of wastage need to be identified within every single activity and process of the foodservice chain identifying its critical points. Reducing food wastage, redistributing unsold or excess food, and recycling/treating FW are important components of waste management strategies towards food service to reduce the economic, social, and environmental impact of FW. For the measures taken to be effective, besides identifying all the FW parameters it is necessary to make strict controls to ensure sustainable production.

Research to identify the influential aspects of the environment, society, and economy around FW is fundamental to enable controlling the impacts generated in the short and long term. The management of well-performed food services, with proper planning, acquisition control in the acquisition of goods, receipt, and storage, can significantly reduce waste, consequently, with less impact on the three main pillars of sustainability. Therefore, more research is needed to address procedures for monitoring FW generation in each type of foodservice activity and find out methods for reducing it in any single situation. This review does not intend to exhaust the subject about the impacts of FW in the three main sustainability dimensions but to open the opportunity for debate and future studies that enable the development of tools to assess the impact of FW from restaurants on global sustainability.

## Data Availability

The study did not report any data.
